# Diabetes and Adverse Reproductive Outcomes in a Group of Mongolian Women: A Comparative Study with Non-Diabetic Subjects

**DOI:** 10.3390/jcm14176344

**Published:** 2025-09-08

**Authors:** Bolor-Erdene Sarankhuu, Enkhjin Gantsolmon, Khangai Enkhtugs, Yanjmaa Sankhuu, Chantsaldulam Purevdorj, Seong-Lan Yu, Seok-Rae Park, Oyuntugs Byambasukh, Jaeku Kang

**Affiliations:** 1 Priority Research Center, Myunggok Medical Research Institute, College of Medicine, Konyang University, Daejeon 35365, Republic of Korea; bolorerdene@konyang.ac.kr (B.-E.S.); yusl73@konyang.ac.kr (S.-L.Y.); srpark@konyang.ac.kr (S.-R.P.); 2 Department of Endocrinology, School of Medicine, Mongolian National University of Medical Sciences, Ulaanbaatar 14210, Mongolia; pmh21d289@st.mnums.edu.mn; 3 English Medical Program, School of Medicine, Mongolian National University of Medical Sciences, Ulaanbaatar 14210, Mongolia; 4 Department of Family Medicine, School of Medicine, Mongolian National University of Medical Sciences, Ulaanbaatar 14210, Mongolia; khangai@mnums.edu.mn; 5 Department of Endocrinology and Diabetes, First State Central Hospital, Ulaanbaatar 14210, Mongolia; s.yanjmaa@fchm.edu.mn (Y.S.); p.chantsaldulam@fchm.edu.mn (C.P.); 6 Department of Microbiology, College of Medicine, Konyang University, Daejeon 35365, Republic of Korea; 7 Department of Pharmacology, College of Medicine, Konyang University, Daejeon 35365, Republic of Korea

**Keywords:** diabetes mellitus, reproductive outcomes, miscarriage, abortion

## Abstract

**Background/Objectives:** Diabetes mellitus (DM) poses an increasing burden in Mongolia, yet its impact on reproductive outcomes remains underexplored. This study aimed to compare pregnancy outcomes between diabetic and non-diabetic women and assess whether diabetes duration influences adverse reproductive events. **Methods:** We conducted a cross-sectional study among 223 diabetic and 495 non-diabetic women attending outpatient clinics in Ulaanbaatar between October and December 2024. Data on reproductive history were collected using structured questionnaires. Pregnancy outcomes included miscarriage, stillbirth, abortion, and live birth. Logistic regression models were applied to assess associations, adjusting for age, marital status, education, smoking, alcohol use, age at menarche, and reproductive history. **Results:** Mean age was 51.7 and 50.4 years for diabetic and non-diabetic women, respectively (*p* = 0.222). Diabetic women had more pregnancies (median: 4.00 vs. 3.00, *p* < 0.001) and a higher likelihood of abortion (35.4% vs. 25.5%, *p* = 0.004) and miscarriage (27.8% vs. 11.1%, *p* < 0.001). Stillbirths were more frequent in diabetic (4.0% vs. 2.2%) but not statistically significant. Pregnancy problems (miscarriage and/or stillbirth) were more prevalent in diabetic women (29.6% vs. 12.7%, *p* < 0.001). In adjusted models, diabetes was associated with higher odds of pregnancy problems (aOR = 1.64, 95% CI: 1.02–2.63, *p* = 0.042), miscarriage (aOR = 2.03, 95% CI: 1.21–3.40, *p* = 0.007), and abortion (aOR = 1.58, 95% CI: 1.14–2.19, *p* = 0.006). A dose response pattern was observed: miscarriage risk was higher in women with diabetes ≥10 years (OR = 2.67, 95% CI: 1.55–4.62, *p* < 0.001) than <10 years (OR = 1.79, 95% CI: 1.08–2.96, *p* = 0.023). **Conclusions:** Diabetes is independently associated with increased risks of miscarriage and abortion in Mongolian women, with longer disease duration further elevating this risk.

## 1. Introduction

Diabetes mellitus (DM) is a rising global health burden, with 537 million adults affected in 2021, a figure expected to reach 783 million by 2045 [1]. While its systemic complications—particularly in cardiovascular and renal systems—are well recognized, less attention has been given to its impact on reproductive outcomes. This gap is particularly critical for women of reproductive age, especially in low- and middle-income countries where healthcare access and continuity of care are limited.

Several clinical and biological mechanisms suggest that diabetes may compromise pregnancy outcomes. Chronic hyperglycemia induces oxidative stress and endothelial dysfunction, which can impair placental development and embryo implantation [2,3,4,5,6,7,8,9]. Insulin resistance and obesity, common in type 2 diabetes, further disrupt ovarian function and endometrial receptivity [2,9,10,11,12]. These physiological disruptions are associated with higher risks of miscarriage, stillbirth, and abortion among diabetic women.

Importantly, pregnancy loss itself—whether related to diabetes or not—remains a substantial global concern. Miscarriage affects approximately 15% of recognized pregnancies, totaling an estimated 23 million annually, while stillbirths account for another 2.6 million third-trimester losses each year [13,14]. These events have consequences beyond immediate loss, including increased risks of future obstetric complications and long-term health effects. Despite their magnitude, pregnancy losses are often underreported and insufficiently prioritized in global health strategies, especially in low-resource settings.

Mongolia, like many rapidly urbanizing nations, has experienced a sharp rise in the prevalence of type 2 diabetes, driven by lifestyle changes and demographic shifts [15,16]. National screening efforts have revealed that women of reproductive age represent an increasing share of the diabetic population [16]; however, data on how diabetes affects pregnancy outcomes in Mongolia remain scarce. Most existing evidence originates from high-income settings and may not be applicable to Mongolian women, given differences in reproductive patterns, healthcare systems, and cultural contexts.

To address this knowledge gap, the present study aimed to examine the association between diabetes and adverse reproductive outcomes among Mongolian women. Specifically, we compared the prevalence of miscarriage, abortion, stillbirth, and live birth between diabetic and non-diabetic women, and evaluated whether the duration of diabetes influenced these risks.

## 2. Materials and Methods

### 2.1. Study Design and Participants

This cross-sectional, comparative study was conducted at the Endocrinology outpatient clinic of the Mongolia–Japan Hospital (MJH) between October and December 2024. Women with a confirmed diagnosis of diabetes mellitus were consecutively recruited during this period. To form the non-diabetic comparison group, women without diabetes were recruited during the same period from the outpatient clinics of both the MJH and the First State Central Hospital. The sampling strategy included age-matching to minimize confounding and reduce selection bias between the diabetic and non-diabetic groups.

Sample size calculations were based on detecting a difference in miscarriage prevalence between diabetic and non-diabetic women (assuming miscarriage rates of 15% in non-diabetic women and 25% in diabetic women based on the existing literature [13,14]), with 80% power and a 5% significance level. A 1:2 case-to-control ratio was selected to increase analytical power and maintain feasibility of recruitment within the defined study period. This yielded a required sample of 250 diabetic and 500 non-diabetic women. A total of 842 eligible women were contacted, with 750 (89.3%) agreeing to participate.

To reduce heterogeneity, we excluded women with a history of gestational diabetes mellitus (GDM) and those with unclear diabetes classification; women with current GDM without pre-existing diabetes were not eligible. Nearly all diabetic participants had clinical characteristics consistent with type 2 diabetes, the most prevalent form in Mongolia. Eligible participants were women of reproductive age with a known diabetic or non-diabetic status who had experienced at least one pregnancy. Women who had never been pregnant or who had known reproductive disorders, such as polycystic ovary syndrome or congenital uterine anomalies, were excluded. After applying these criteria, a total of 223 diabetic and 495 non-diabetic women were included in the final analysis. All participants provided information on their demographic background, clinical profile, and reproductive history through structured interviews conducted by the research team.

### 2.2. Reproductive History and Outcome Definitions

Each participant completed a single interview lasting 20–30 min, conducted in a private room by a trained physician. The reproductive history questionnaire included 28 items (20 closed-ended and 8 open-ended), adapted from WHO tools and pilot-tested in 30 Mongolian women for cultural appropriateness and clarity. Reproductive history was recorded using a questionnaire that captured the number of pregnancies, live births, miscarriages, stillbirths, and medically induced abortions. Pregnancy was defined as any confirmed conception regardless of gestational age. A live birth referred to the delivery of an infant who exhibited any signs of life after 24 weeks of gestation. Miscarriage was defined as spontaneous loss of pregnancy before the 20th week of gestation. Stillbirth was defined as intrauterine fetal death occurring at or beyond 24 weeks of gestation without any signs of life at delivery [17,18]. Medically induced termination of pregnancy at any gestational age was classified as abortion. For the purposes of analysis, a composite outcome termed “pregnancy problem” was defined to include either miscarriage or stillbirth. Abortion was examined as a separate outcome and was not included in this composite.

### 2.3. Diabetes-Related Data

Diagnosis of diabetes mellitus was based on national clinical criteria aligned with WHO guidelines, confirmed by medical records (e.g., FPG ≥ 7.0 mmol/L or HbA1c ≥ 6.5%). Among diabetic participants, additional data were collected on duration since diagnosis, current treatment modalities (including use of oral glucose-lowering agents or insulin), and presence of chronic complications such as retinopathy, nephropathy, or neuropathy. Diabetes duration was categorized as less than 10 years or 10 years or longer. This categorization was used to evaluate whether the length of disease duration was associated with reproductive outcomes.

### 2.4. Covariates and Confounders

Demographic and lifestyle characteristics, including age, body mass index (BMI), educational level, marital status, smoking status, and alcohol use, were collected for all participants. BMI was calculated using measured height and weight and categorized according to the World Health Organization classification. Education was grouped into lower (up to high school), middle (college or vocational school), and higher (university degree or above). Marital status was recorded as cohabitant (living with a partner) or non-cohabitant. Smoking status and alcohol use were based on a self-report. Smoking was categorized as current smoker or non-smoker, while alcohol use was categorized as regular drinker (defined as consumption at least once per week) or non-drinker. Age at menarche, total number of pregnancies, and total number of live births were also recorded and treated as continuous variables in adjusted models.

### 2.5. Statistical Analysis

Descriptive statistics were used to summarize participant characteristics by diabetes status. Continuous variables were expressed as means and standard deviations or as medians with interquartile ranges, depending on distribution. Categorical variables were presented as frequencies and percentages. Group differences were assessed using independent-sample *t*-tests or Mann–Whitney U tests for continuous variables, and chi-square tests for categorical variables.

Logistic regression was used to evaluate the association between diabetes and the following outcomes: pregnancy problems (miscarriage and/or stillbirth), miscarriage alone, and abortion. For each outcome, odds ratios (ORs) and 95% confidence intervals (CIs) were reported. The models were constructed in a stepwise fashion. Model 1 adjusted for age. Model 2 additionally adjusted for education level, marital status, smoking, and alcohol use. Model 3 further included age at menarche, number of pregnancies, and number of live births. Model 4 included all previous variables and additionally adjusted for body mass index (BMI). In other words, each model incrementally accounted for a broader range of potential confounders to evaluate the robustness of associations between diabetes and adverse reproductive outcomes. An exploratory analysis was also conducted using diabetes duration as a three-level exposure variable (non-diabetic, diabetic < 10 years, diabetic ≥ 10 years) to examine a potential dose–response association with adverse reproductive outcomes. The 10-year cutoff was based on previous studies identifying increased microvascular and reproductive risks beyond this duration. This classification also ensured adequate sample sizes within each subgroup. We used 45 years as a cutoff to reflect the upper limit of reproductive age and ensure adequate subgroup sizes; sensitivity analysis using a 35-year threshold yielded comparable trends.

Adjusted estimated means of the number of pregnancy problems across diabetes duration groups were obtained using analysis of covariance (ANCOVA), controlling for all previously mentioned covariates. Interaction terms were tested to explore whether age or diabetes duration modified the associations, but no significant interactions were found.

All statistical analyses were performed using IBM SPSS Statistics, version 28.0 (IBM Corp., Armonk, NY, USA). A two-sided *p*-value less than 0.05 was considered statistically significant.

## 3. Results

### 3.1. General and Reproductive Characteristics

**Figure 1:**
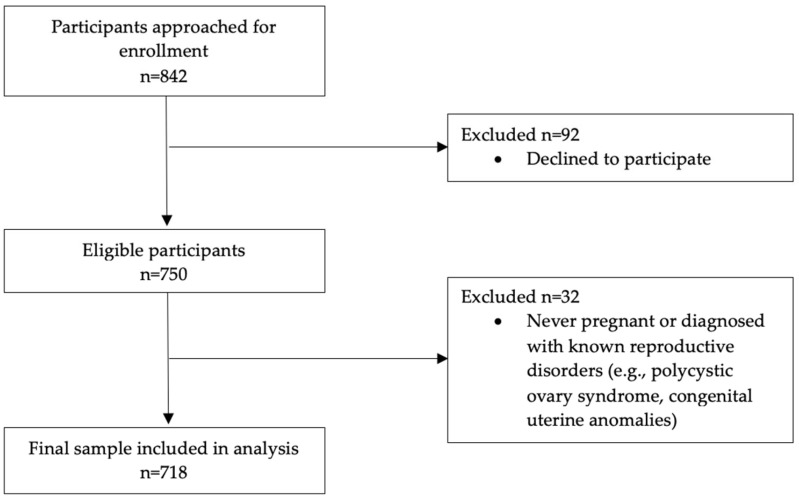
Flow diagram of participant recruitment and inclusion.

A total of 842 women were approached for participation in the study. Of these, 92 declined to participate, and 32 were excluded due to never having been pregnant or having a known reproductive disorder. The final analytic sample consisted of 718 women, including 223 with diabetes and 495 without diabetes. The recruitment process is illustrated in the participant flow diagram (Figure 1). Table 1 summarizes the general and reproductive characteristics of the study population by diabetes status. The mean age was slightly higher in the diabetic group (51.70 ± 13.9 years) compared to the non-diabetic group (50.39 ± 13.0 years), although this difference was not statistically significant (*p* = 0.222). The distribution of age categories, BMI, educational level, marital status, alcohol consumption, and smoking was similar between groups.

In terms of reproductive health, age at menarche was similar between groups (14.18 ± 2.45 vs. 14.00 ± 1.74 years, *p* = 0.282). The number of pregnancies was higher among diabetic women (median 4.00, IQR: 2.00–6.00) compared to non-diabetic women (median 3.00, IQR: 2.00–4.00; *p* < 0.001). The number of live births was also slightly higher in diabetic women (median 3.00, IQR: 2.00–4.00) than in non-diabetic women (median 3.00, IQR: 2.00–4.00; *p* = 0.061).

Regarding abortions, both groups had a median of zero, reflecting skewed distributions; however, diabetic women had a significantly higher likelihood of having had at least one abortion (35.4% vs. 25.5%, *p* = 0.004).

### 3.2. Pregnancy-Related Problems

As shown in Table 2, diabetic women reported significantly more pregnancy-related problems—defined as miscarriage and/or stillbirth—than non-diabetic women (29.6% vs. 12.7%, *p* < 0.001). Miscarriage was the major contributor to this difference. A significantly higher proportion of diabetic women experienced at least one miscarriage (27.8% vs. 11.1%, *p* < 0.001). However, due to the skewed distribution and median value of zero in both groups, the number of miscarriages was not reported as a central tendency measure in the table. Stillbirths were also more common among diabetic women (4.0% vs. 2.2%), although the difference did not reach statistical significance (*p* = 0.132). As with miscarriage, the median number of stillbirths was zero in both groups.

### 3.3. Pregnancy Problems by Diabetes Duration

To explore the impact of diabetes duration on pregnancy outcomes, diabetic women were stratified into two groups: <10 years and ≥10 years since diagnosis. The median number of pregnancy problems (miscarriage and/or stillbirth) was slightly higher in women with longer diabetes duration, though the difference was not statistically significant (median: 0.00 [IQR: 0.00–1.00] vs. 0.00 [0.00–1.00], *p* = 0.145). Miscarriage was the main contributor, with a significantly higher median number in those with diabetes duration ≥10 years (0.00 [0.00–1.00]) compared to <10 years (0.00 [0.00–0.00], *p* = 0.006). Stillbirth remained uncommon across both groups and did not differ significantly by diabetes duration (*p* = 0.648).

**Figure 2:**
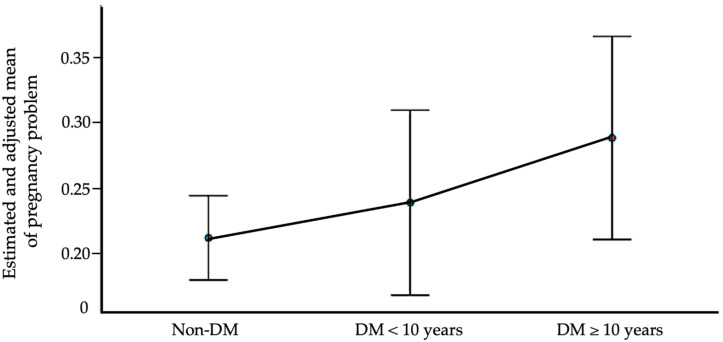
Adjusted mean number of pregnancy problems by diabetes status and duration. Notes: Model-based adjusted means were estimated using a generalized linear model controlling for age, education, marital status, smoking, alcohol use, age at menarche, number of pregnancies, and number of live births. Pregnancy problems include miscarriage and stillbirth. The error bars represent 95% confidence intervals.

Due to the skewed distribution of outcomes, model-based adjusted means were also estimated using a generalized linear model. In this analysis, non-diabetic women were included as a reference group to assess the potential dose–response relationship with diabetes duration. After adjusting for age, education, marital status, smoking, alcohol use, age at menarche, number of pregnancies, and live births, the adjusted mean number of pregnancy problems increased progressively: 0.218 (95% CI: 0.179–0.256) in non-diabetic women, 0.288 (95% CI: 0.219–0.357) in women with diabetes <10 years, and 0.366 (95% CI: 0.309–0.423) in those with diabetes ≥10 years (Figure 2). This pattern supports a possible dose–response association between diabetes duration and adverse pregnancy outcomes.

### 3.4. Regression Analysis of Adverse Pregnancy Outcomes in Women with Diabetes

Table 3 presents the results from logistic regression models evaluating the association between diabetes and adverse pregnancy outcomes. In the unadjusted model, diabetes was significantly associated with increased odds of experiencing a pregnancy problem (OR = 2.354, 95% CI: 1.644–3.370, *p* < 0.001). This association remained statistically significant after adjusting for age (Model 1), sociodemographic factors (Model 2), and reproductive history (Model 3), with an adjusted odds ratio of 1.752 (95% CI: 1.052–2.919, *p* = 0.031) and Model 4 additionally included body mass index (BMI) to assess its potential confounding influence. The final model (Model 4) showed that diabetes was still significantly associated with pregnancy problems (OR = 1.701, 95% CI: 1.021–2.863, *p* = 0.001). Pregnancy problems is a composite variable including miscarriage or stillbirth; miscarriage was also analyzed individually due to higher frequency. Similarly, the odds of miscarriage were consistently higher among women with diabetes across all models. In the fully adjusted model (Model 3), the odds ratio for miscarriage was 2.026 (95% CI: 1.208–3.399, *p* = 0.007). In the fully adjusted model including BMI (Model 4), the odds ratio for miscarriage was 2.038 (95% CI: 1.113–4.184, *p* = 0.001), confirming a strong independent association between diabetes and miscarriage even after adjusting for a wide range of potential confounders.

To further explore the role of diabetes chronicity, women were stratified into three groups based on diabetes status and duration: non-diabetic, diabetic <10 years, and diabetic ≥10 years. Compared to non-diabetic women, those with diabetes for <10 years had a modest, non-significant increase in the odds of pregnancy problems (OR = 1.432, 95% CI: 0.934–2.195, *p* = 0.101), while those with diabetes ≥10 years had significantly higher odds (OR = 2.034, 95% CI: 1.318–3.139, *p* = 0.002). A similar dose–response pattern was observed for miscarriage. Women with diabetes <10 years had 1.79 times higher odds of miscarriage (95% CI: 1.082–2.957, *p* = 0.023), and those with ≥10 years had 2.67 times higher odds (95% CI: 1.547–4.616, *p* < 0.001). These findings suggest that longer diabetes duration is independently associated with greater reproductive risk.

In an additional model examining abortion, diabetes was found to be a significant predictor. After controlling for sociodemographic and reproductive factors, diabetic women had significantly higher odds of having experienced at least one abortion (OR = 1.578, 95% CI: 1.137–2.191, *p* = 0.006). Although abortion was not included in the composite pregnancy problem outcome, this result highlights another dimension of reproductive burden linked to diabetes.

### 3.5. Miscarriage Patterns by Age and Diabetes Duration

To further explore the combined influence of diabetes duration and reproductive potential, we stratified miscarriage data by age group (<45 vs. ≥45 years) and diabetes duration. Since advancing age is naturally associated with reduced fertility and higher pregnancy risks, analyzing age in conjunction with diabetes duration provides a more nuanced understanding.

**Figure 3:**
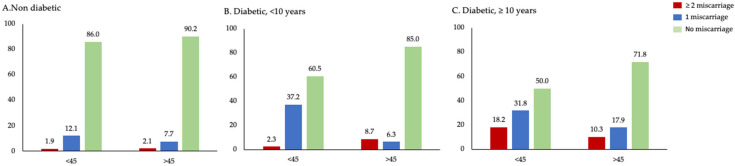
Miscarriage patterns by age group and diabetes duration. Notes: Percentage distribution of women experiencing no miscarriage, one miscarriage, or two or more miscarriages, stratified by age group (<45 years vs. ≥45 years) and diabetes status: (**A**) non-diabetic, (**B**) diabetic <10 years, and (**C**) diabetic ≥10 years. The proportion of miscarriage was notably higher in diabetic women, especially those with longer diabetes duration and younger age.

Figure 3 shows that miscarriage was notably more frequent among diabetic women, particularly those with a longer duration of diabetes. Among women aged <45 years with diabetes ≥10 years, 18.2% had two or more miscarriages and 31.8% had one, compared to just 1.9% and 12.1%, respectively, in non-diabetic counterparts. In the ≥45 age group, the same pattern was observed: women with long-standing diabetes had the highest prevalence of multiple miscarriages (10.3%), while non-diabetics mostly reported no history of miscarriage (90.2%).

## 4. Discussion

This study provides new insight into the reproductive consequences of diabetes in a group of Mongolian women, a population underrepresented in the global literature. However, due to the cross-sectional design and the use of a hospital-based sample from urban clinics, the findings cannot be generalized to the entire Mongolian population. Our findings show that diabetes mellitus is significantly associated with increased risks of miscarriage and abortion, even after adjusting for sociodemographic and reproductive confounders. Additionally, we observed a dose–response pattern in which a longer duration of diabetes was associated with higher risk of miscarriage, supporting the notion that chronic metabolic dysregulation may adversely affect reproductive health.

Our findings are broadly consistent with the prior international literature demonstrating an increased risk of adverse pregnancy outcomes among women with diabetes [19,20,21,22,23,24]. Shani et al. conducted a systematic review and meta-analysis and reported significantly elevated risks of miscarriage (OR 1.57) and stillbirth (OR 1.48) in diabetic women compared to non-diabetic counterparts [2]. Similarly, a national cohort study from the United Kingdom by McGrogan et al. found that both type 1 and type 2 diabetes were associated with higher rates of miscarriage and perinatal complications [22]. Consistent with these results, our study found nearly twofold higher adjusted odds of miscarriage among diabetic women (OR = 2.03), with an even greater risk observed among those with a diabetes duration of ≥10 years (OR = 2.67). Additional studies further support these associations. A retrospective study from Saudi Arabia reported increased risks of cesarean section, macrosomia, preterm delivery, and low APGAR scores among women with pregestational diabetes mellitus (PDM), along with a 2.6-fold higher stillbirth rate, although the latter did not reach statistical significance due to limited sample size [23]. A study also revealed a higher rate of miscarriage among women with type 1 diabetes treated with continuous subcutaneous insulin infusion, suggesting that disease severity and insulin delivery modality may influence outcomes [24].

Mechanistically, several pathways may underline these associations. Hyperglycemia in early gestation can induce oxidative stress, endothelial dysfunction, and epigenetic changes that impair embryo implantation and placental development [2,3,4,5,6]. Studies have also shown that long-standing diabetes is associated with impaired endometrial receptivity and suboptimal uterine perfusion, both of which increase the risk of pregnancy loss [8,9]. Moreover, women with diabetes are at elevated risk for vascular and immunologic complications that can further compromise reproductive outcomes [9,10]. Moreover, Chakrabarti et al. investigated hormonal and metabolic profiles in 200 Indian women with type 2 diabetes and found a significant association between miscarriage and elevated BMI, fasting glucose, and altered levels of reproductive hormones (LH, FSH, insulin, testosterone) [25,26]. Their study underscores the complex hormonal milieu associated with reproductive failure in diabetic women, pointing to metabolic and endocrine disruption as a possible mechanism. In a large population-based study from Norway, type 2 diabetes was associated with a 38% higher risk of miscarriage after adjustment for age, alongside other cardiometabolic conditions such as atherosclerosis and hypertension [27]. These population-based results reinforce the plausibility of a pathophysiological link between metabolic dysfunction and early pregnancy loss.

Interestingly, despite this consistent association with miscarriage, we did not observe a statistically significant difference in stillbirth rates between diabetic and non-diabetic women in our study. This discrepancy may be attributed to the relatively low number of stillbirth events, limiting statistical power. Nonetheless, as emphasized in a recent review, preexisting diabetes increases maternal and fetal metabolic stress, heightening the risk of organogenesis defects, fetal growth restriction, and complications such as nephropathy and retinopathy [28]. These findings highlight the importance of preconception care, tight glycemic control, and early pregnancy monitoring to reduce the risk of adverse outcomes.

Additionally, our findings on abortion—defined as induced termination—highlight another dimension of reproductive burden. Diabetic women had 1.6 times greater odds of reporting abortion compared to non-diabetic women. Although we lacked data on reasons for abortion, prior studies suggest that poor glycemic control, unplanned pregnancies, or fetal anomaly concerns often influence such decisions [29,30]. These underscore the need for comprehensive reproductive counseling integrated into diabetes management.

A key strength of our study is that it addresses a critical knowledge gap in Mongolia, where the reproductive consequences of diabetes remain underexplored. Our relatively large sample and inclusion of both duration of diabetes and comprehensive reproductive history enabled a nuanced assessment of risk. However, some limitations must be acknowledged. First, the cross-sectional design prevents causal inference, and recall bias may have affected the self-reported pregnancy outcomes. Second, clinical parameters such as HbA1c or glucose levels were not available, precluding assessment of glycemic control. Third, although we adjusted for key confounders, unmeasured factors such as socioeconomic status, nutritional status, and quality of antenatal care may still influence the observed associations. Fourth, the relatively small number of stillbirths limits statistical power to detect associations in that outcome. Finally, selection bias is possible due to the recruitment of participants from urban outpatient clinics, which may overrepresent women with greater healthcare access. This may result in underestimation or overestimation of certain reproductive risks. Although the response rate was high, selection bias cannot be ruled out, and findings may not fully represent the general population of Mongolian women.

From a public health and clinical perspective, our findings underscore the importance of integrating reproductive counseling into routine diabetes care. Women of reproductive age with diabetes should receive preconception counseling, targeted contraceptive support, and close monitoring throughout pregnancy. In Mongolia, where access to specialized endocrinology and obstetric services remains limited, especially in rural regions, strengthening primary care systems and referral pathways is critical. Furthermore, future research should employ longitudinal designs to establish temporal relationships between diabetes onset, glycemic control, and reproductive events. Incorporating markers such as HbA1c, inflammatory markers, and uterine Doppler imaging could further elucidate mechanisms. Moreover, qualitative studies exploring women’s perspectives on abortion and pregnancy loss in the context of diabetes would provide valuable context for developing culturally sensitive interventions.

## 5. Conclusions

In conclusion, our findings indicate that diabetes is a significant and independent risk factor for miscarriage and abortion, with longer disease duration amplifying these risks. These results reinforce the need for early diabetes detection, effective management, and coordinated reproductive care. Future longitudinal studies are warranted to confirm causality and assess the impact of interventions on reproductive outcomes in this population.

## Figures and Tables

**Table 1:** Characteristics of study population.

Findings	Diabetic (*n* = 223)	Non-Diabetic (*n* = 495)	*p* Value
Age (years)	51.70 ± 13.9	50.39 ± 13.0	0.222
Age group, % (*n*)			
<45 years	29.1 (65)	31.7 (157)	0.541
≥45 years	70.9 (158)	68.3 (338)	
Diabetes duration	9.1 (9.0)	-	-
Body mass index (kg/m^2^)	24.65 ± 4.6	24.55 ± 2.9	0.733
Body mass index category, % (*n*)			
Normal weight	63.2 (141)	62.2 (308)	0.367
Overweight	28.7 (64)	32.1 (159)	
Obesity	8.1 (18)	5.7 (28)	
Education, % (*n*)			
Lower	5.9 (13)	3.0 (15)	0.200
Middle	36.0 (80)	44.0 (218)	
Higher	58.1 (130)	52.9 (262)	
Marital status, % (*n*)			
Cohabitant	87.9 (196)	88.5 (438)	0.621
Non-cohabitant	12.1 (27)	11.5 (57)	
Alcohol use, % (*n*)	5.9 (13)	5.9 (29)	0.726
Smoking, % (*n*)	4.0 (9)	7.3 (36)	0.259
Age at first menstruation (years)	14.18 ± 2.45	14.00 ± 1.74	0.282
Number of pregnancies	4.0 (2.0–6.0)	3.0 (2.0–5.0)	<0.001
Live birth			
Number of live births	3.0 (2.0–4.0)	3.0 (2.0–4.0)	0.061
≥1 Live birth, % (*n*)	98.2% (219)	98.0% (485)	0.549
Abortion			
≥1 Abortion, % (*n*)	35.4% (79)	25.5% (126)	0.004

Notes: Data are presented as mean ± SD or median (interquartile ranges) and percentages (numbers).

**Table 2:** Comparative reproductive outcomes between diabetic and non-diabetic women.

Findings	Diabetic (*n* = 223)	Non-Diabetic (*n* = 495)	*p* Value
Pregnancy problems			
≥1 Pregnancy problem, % (*n*)	29.6 (66)	12.7 (61)	<0.001
Miscarriage			
≥1 Miscarriage, % (*n*)	27.8 (62)	11.1 (55)	<0.001
Stillbirth			
≥1 Stillbirth, % (*n*)	4.0 (9)	2.2 (11)	0.132

Notes: Data are presented as percentages (numbers).

**Table 3:** Logistic regression analysis of reproductive outcomes in diabetic versus non-diabetic women.

Findings	Logistic Regression		
	Odds Ratio	95% CI	*p* Value
**Pregnancy problem**			
Crude	2.187	1.517; 3.154	<0.001
Model 1	2.275	1.570; 3.296	<0.001
Model 2	2.253	1.552; 3.273	<0.001
Model 3	1.638	1.019; 2.631	0.042
Model 4	1.701	1.021; 2.863	0.001
**Miscarriage**			
Crude	2.895	1.893; 4.427	<0.001
Model 1	2.988	1.947; 4.586	<0.001
Model 2	2.961	1.926; 4.552	<0.001
Model 3	2.026	1.208; 3.399	0.007
Model 4	2.038	1.113; 4.184	0.001

Notes: Logistic regression analysis shows the odds ratio with a 95% CI. The dependent variable is miscarriage occurrence, and the independent variable is diabetes status compared to non-diabetic women. Model 1: Adjusted for age. Model 2: Adjusted for age, marital status, education, smoking, and alcohol. Model 3: Adjusted for age, marital status, education, smoking, alcohol, number of pregnancies, number of live births, and menarche age. Model 4: Adjusted for age, marital status, education, smoking, alcohol, number of pregnancies, number of live births, menarche age, and BMI.

## Data Availability

The data used to support the findings of this study are available from the corresponding author upon request.
